# Modification of Surfaces with Vaterite CaCO_3_ Particles

**DOI:** 10.3390/mi13030473

**Published:** 2022-03-19

**Authors:** Bushra Zafar, Jack Campbell, Jake Cooke, Andre G. Skirtach, Dmitry Volodkin

**Affiliations:** 1 Department of Chemistry and Forensics, School of Science and Technology, Nottingham Trent University, Nottingham NG11 8NS, UK; bushra.zafar2020@my.ntu.ac.uk (B.Z.); jack.campbell@ntu.ac.uk (J.C.); jake.cooke2018@my.ntu.ac.uk (J.C.); 2 Nanotechnology Laboratory, Faculty of Bioscience Engineering, Ghent University, 9000 Ghent, Belgium; andre.skirtach@ugent.be

**Keywords:** crystal growth, calcium carbonate, immobilization, biomineralization

## Abstract

Former studies have demonstrated a strong interest toward the crystallization of CaCO_3_ polymorphs in solution. Nowadays, CaCO_3_ crystallization on solid surfaces is extensively being studied using biomolecules as substrates for the control of the growth aiming at various applications of CaCO_3_. Calcium carbonate exists in an amorphous state, as three anhydrous polymorphs (aragonite, calcite and vaterite), and as two hydrated polymorphs (monohydrocalcite and ikaite). The vaterite polymorph is considered as one of the most attractive forms due to its large surface area, biocompatibility, mesoporous nature, and other features. Based on physical or chemical immobilization approaches, vaterite can be grown directly on solid surfaces using various (bio)molecules, including synthetic polymers, biomacromolecules such as proteins and peptides, carbohydrates, fibers, extracellular matrix components, and even biological cells such as bacteria. Herein, the progress on the modification of solid surfaces by vaterite CaCO_3_ crystals is reviewed, focusing on main findings and the mechanism of vaterite growth initiated by various substances mentioned above, as well as the discussion of the applications of such modified surfaces.

## 1. Introduction

In nature, a variety of organisms use biomolecules with functionalization to induce biomineralization and crystallization of calcium carbonate polymorphs with controlled size, texture, and morphology [1,2]. Evidently, the formed calcium carbonate crystals have diverse properties such as varying stability, solubility, the potential controlled release of any contained biomolecules, and the modification of surfaces of biomolecular substrates [3]. Former studies have shown that vaterite can be synthesized in solution containing biomolecules, such as proteins, polypeptides and polysaccharides, and is now being immobilized on the surface of biomolecules, as the acidic segments of these biomolecules act as growth modulators. The biomolecules have strong binding to certain mineral polymorphs of calcium carbonates, which in turn favours the formation of vaterite by inhibiting synthesis of other polymorphs. The biomolecules, which are to be incorporated into calcium carbonate, deprotonate acidic groups on their surface at alkaline pH with more affinity towards certain calcium carbonate crystal planes, leading to vaterite formation at such surfaces [4].

Generally, calcium carbonate solidifies into three anhydrous polymorphic forms including aragonite, calcite and vaterite. Two hydrated crystalline polymorphs are also known, namely monohydrocalcite (CaCO_3_·H_2_O) and ikaite (CaCO_3_·6H_2_O); besides this, CaCO_3_ may also exist in its amorphous form [5,6]. Vaterite is the rarest form of anhydrous CaCO_3_ due to its inherently low stability and the tendency to re-crystallise into the more thermodynamically stable polymorph—calcite. The vaterite polymorph is, however, a naturally derived mineral that can be found in nature or synthesized in a laboratory setting using simple methods [7], such as via simple mixing methods [8]. Simultaneously, vaterite is an alluring polymorph of CaCO_3_ in material science studies due to its potential applications in drug delivery, regenerative medicine, and bone implants [9]. The direct superficial crystallization of vaterite is beneficial for the creation of biocompatible surfaces and targeted drug particles [4,10,11,12]. Vaterite is generally prepared in the laboratory using calcium chloride and sodium carbonate [13]. Its vaterite form is more beneficial for research purposes due to its wide range of applications [14]; owing to its inherent biocompatibility and biodegradability [7], as well as the capacity to host large amounts of bioactive compounds due to its mesoporosity [15,16]. Hence, the synthesis of vaterite with controlled size and morphology is recently a major field of interest [17] and will be the polymorph of focus for this review. It is worth mentioning the immobilization of other polymorphs is of great interest, but this topic is out of the scope of this review and will be considered in our future research.

It is widely accepted now that biomolecules can be used as solid templates for the biomineralization of vaterite CaCO_3_. Proteins, peptides and polysaccharides are important growth mediators for the crystallization of vaterite on their surfaces, via assisting in its crystallization. Various proteins, being hydrophobic in nature, also assist in the formation of CaCO_3_ nucleation [18,19]. Former studies engrossed in the design of solid models to inspect the effect of macromolecules on the nucleation and growth of CaCO_3_ crystals as cell membranes of biomolecules that may act as a template for the biomineralization of calcium carbonates [20,21,22,23,24,25,26,27]. Vaterite crystallization on solid substrates is assisted by physical and chemical immobilization approaches as aforementioned [28]. The physical immobilization approach entails the nucleation of vaterite crystals at the expense of electrostatic, hydrogen bonding and other non-covalent interactions, leading to the adsorption of crystalline material on solid surfaces. Where chemical immobilization involves the binding and growth of vaterite crystals onto the solid surfaces via covalent binding [1,29]. The CaCO_3_ crystals have also been used to modify titanium metal surfaces; after having been deposited, the organic system mimics the bone tissue chemical and physical features. It was indicated in former studies that the bioactive and osteoconductive features of vaterite is one of the profuse biominerals present in nature [30].

Despite vaterite crystallization in the presence of various substances being well studied and reported in literature, formation of vaterite onto solid surfaces upon its crystallization is covered only by a rather limited number of studies, so far. These substances include various (bio)molecules like synthetic polymers, biomacromolecules as proteins and peptides, carbohydrates, fibers, extracellular matrix components and even biological cells such as bacteria. Control over the crystallization of vaterite onto a solid surface is still a challenge and the understanding of this process is of high importance in both industry and fundamental research. Herein, this review illustrates the progress in the immobilization of CaCO_3_ vaterite on solid surfaces via chemical and physical processes governed by a number of active compounds mentioned above.

## 2. Modification of Various Surfaces with Vaterite CaCO_3_

### 2.1. Peptide Modified Surfaces

**Figure 1:**
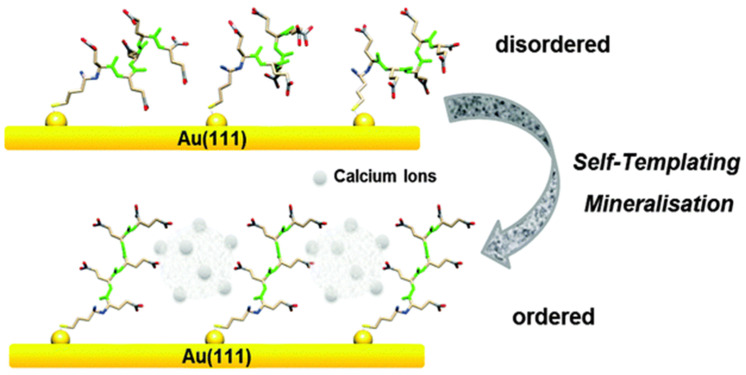
Schematic drawing of Glu5 peptide layer on Au when interacting with CaCO_3_ precursors. This Figure is adapted with permission from [31].

Vaterite can be crystallized onto inorganic surfaces via use of biomolecules such as peptides carrying acidic side groups. The direct immobilization of vaterite can be convenient for the design of biocompatible surfaces; for instance, there is an interaction between surface bound oligo (glutamic acid) polypeptides and CaCO_3_ polymorphs (mainly vaterite). The thiol-terminated oligo (glutamic acid) peptide (Figure 1) attached to a gold substrate acts as a model surface; and when interacting with calcium ions, acts as a template for the precipitation of vaterite. Herein, the structural composition of oligopeptides chemically attached to the gold substrate is the main driving force for the mineralization of vaterite. As a result, a high affinity between the carboxylic groups of the peptide and calcium ions suppresses calcite growth, and favours vaterite formation. Calcium ions bound to the peptide surfaces act as linking agents of which also promotes structural changes in peptide conformation, leading to the formation of vaterite. The formation is due to a “self-templating process” in which calcium favours the formation. Thus, calcium ions modify the oligo (glutamic acid) peptides acting as a precursor, and self-template the vaterite structure [31] (Figure 1).

**Figure 2:**
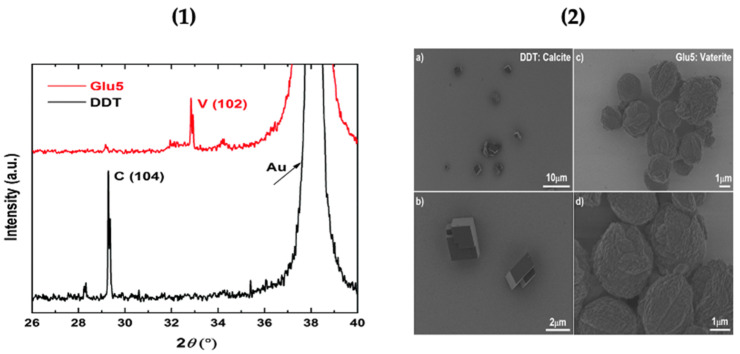
(**1**) XRD pattern of calcium carbonate formed on Glu5 peptide (red) and DDT (black) monolayers on Au substrate. (V) Vaterite; (C) Calcite. (**2**) SEM images of calcium carbonate polymorphs formed on DDT: calcite (**a**,**b**) and Glu5 peptide monolayers: vaterite (**c**,**d**) on Au. This Figure is adapted with permission from [31].

Vaterite formation brought about by the self-templating process discussed above was characterized using x-ray diffraction (XRD). The characterization was done using a reference surface dodecanethiol (DDT) self-assembled monolayer. The biomineralization of CaCO_3_ polymorphs on peptides attached to the gold surface was studied using x-ray diffraction (Figure 2). The 2θ value of 32.8° is assigned to vaterite and confirms its crystallisation and stability on the peptide-coated gold substrate, whilst the reference monolayer DDT favours the formation of calcite (Figure 2). The biomineralization of vaterite on the functionalized gold substrate was further confirmed by scanning electron microscopy (SEM). The spherical vaterite can be seen on the surface, whilst the reference DDT monolayer shows the typical cubic calcite structures on its surface [31] as shown in Figure 2. Hence demonstrating the selective growth of vaterite upon peptide-functionalized surfaces. The next section below evaluates the growth of vaterite upon cellulose surfaces.

### 2.2. Cellulose Surfaces

**Figure 3:**
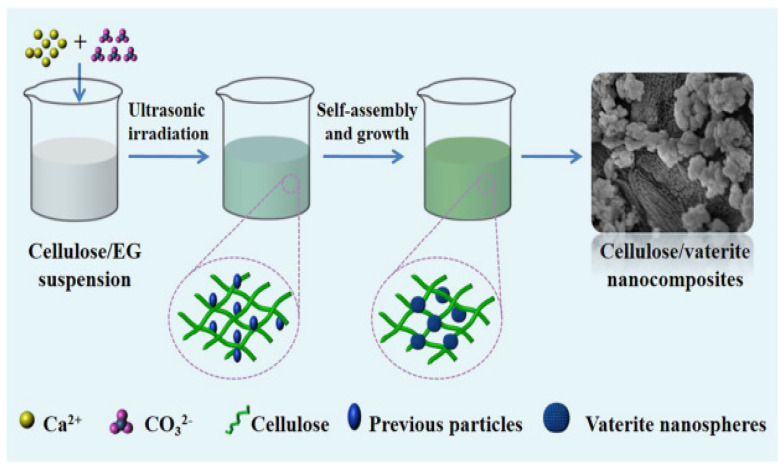
Design for the formulation of cellulose/vaterite nanocomposites by the sono-chemical method. Adapted with permission from [17].

Vaterite immobilization is typically done using a physical immobilization approach, as opposed to chemical approaches. Here, cellulose can be used as an anchor for calcium carbonate nucleation. Cellulose consists of glucose units and is an important biomass present in nature. Cellulose can be employed as a substrate leading to the formation of CaCO_3_ polymorphs on its surface. Cellulose and vaterite nanocomposite materials have excellent cytotoxicity, along with an elevated protein adsorption capacity—ideal for local drug delivery and tissue engineering applications. Results have shown that calcium ions adsorb to the hydroxyl groups along the cellulose backbone; carboxylic groups present on the surface of cellulose also contribute by accumulating calcium ions, leading to the nucleation of CaCO_3_ polymorphs and crystal growth [17] (Figure 3). 

**Figure 4:**
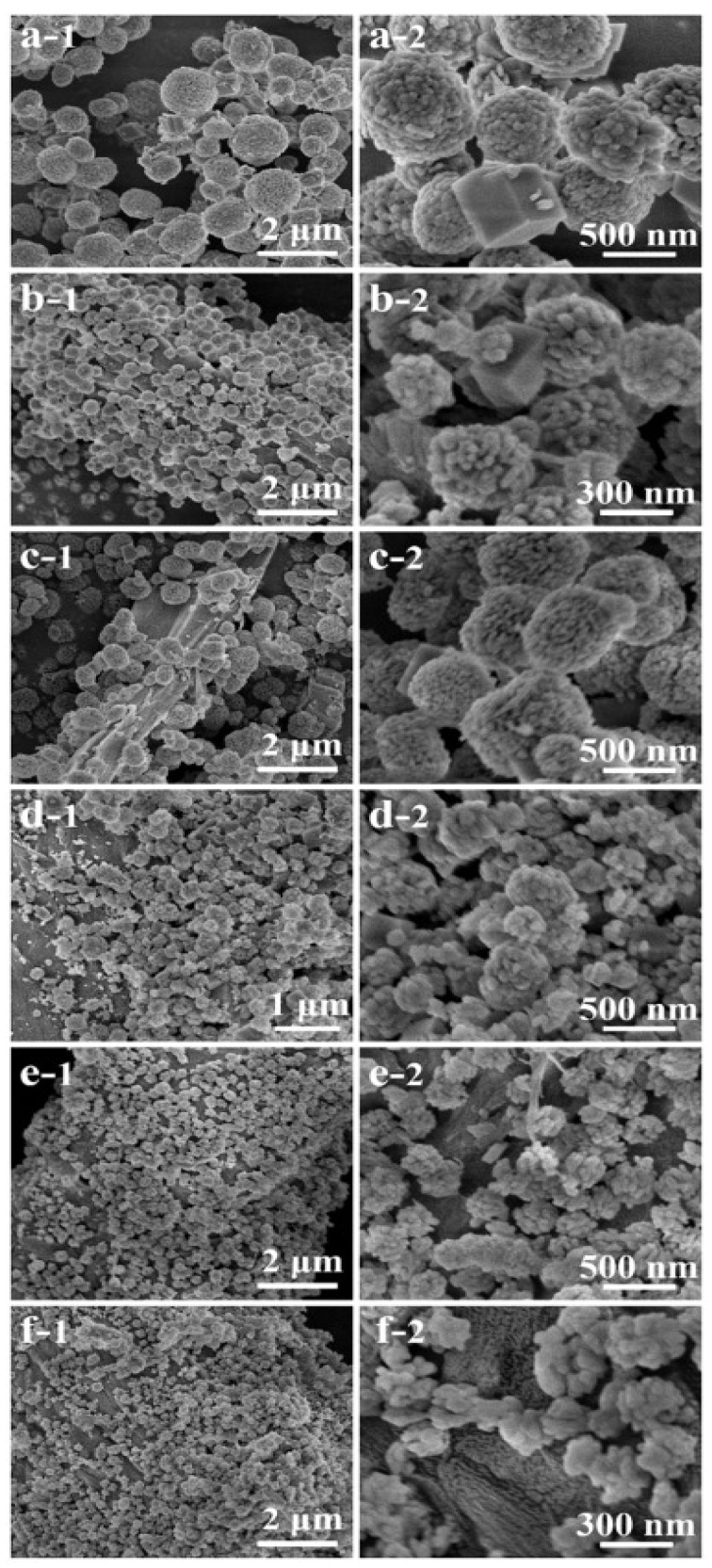
SEM images of the products prepared with different cellulose concentrations: (**a**) the control sample obtained without cellulose; (**b**) 0.51 mg mL^−1^; (**c**) 1.01 mg mL−1; (**d**) 2.03 mg mL^−1^; (**e**) 4.05 mg mL^−1^; (**f**) 8.10 mg mL^−1^. This Figure is adapted with permission from [17].

Studies have shown that cellulose favours the formation of CaCO_3_ polymorphs. XRD and SEM confirmed the formation of vaterite on the surface of cellulose; SEM was used to study the morphologies of prepared vaterite and calcite samples. The samples prepared without cellulose were a mixture of porous vaterite and polyhedral calcite (Figure 4a), whereas in the presence of cellulose, the sizes of vaterite and calcite decreased, and a greater number of crystals were present on the surface (Figure 4b). An increase in cellulose concentration to 1.01 and 2.03 mg/mL resulted in calcite formation (Figure 4c,d). When the concentration of cellulose was increased to 4.05 and 8.10 mg/mL, there was a binding of vaterite onto cellulose, no individual vaterite crystals and calcite formation was suppressed (Figure 4e,f). The image F-2 shows that rice-shaped nanoparticles are formed from vaterite nanospheres [17].

**Figure 5:**
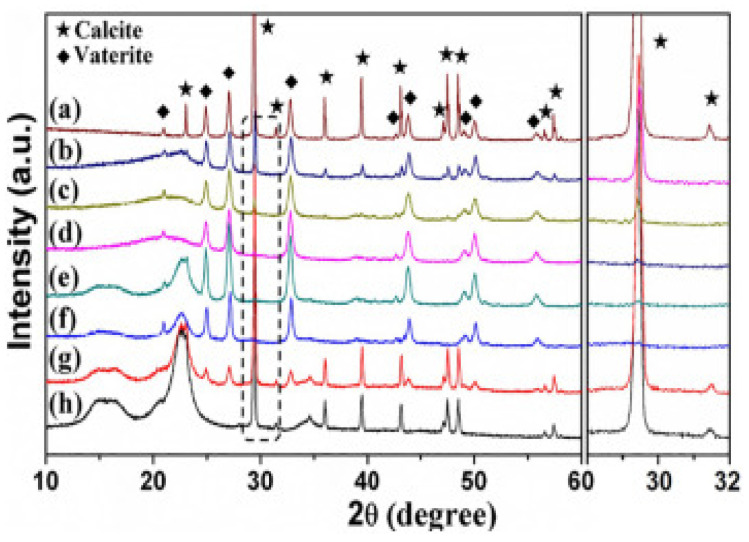
XRD patterns of the products prepared with different cellulose concentrations: (**a**) the control sample obtained without cellulose; (**b**) 0.51 mg mL^−1^; (**c**) 1.01 mg mL^−1^; (**d**) 2.03 mg mL^−1^; (**e**) 4.05 mg mL^−1^; (**f**) 8.10 mg mL^−1^; (**g**) 16.20 mg mL^−1^; (**h**) 32.40 mg mL^−1^. Taken with permission from [17].

XRD (Figure 5) demonstrated that vaterite and calcite crystals formed without cellulose were hexagonal and rhombohedral, whereas the presence of cellulose suppresses calcite formation leading to the formation of more vaterite crystals at 2θ = 22.6°. By increasing cellulose concentration further, calcite crystallization was inhibited completely and vaterite was formed. Hence, cellulose plays a crucial role during the crystallisation of CaCO_3_ polymorphs, and controlled abundancies of both calcite and vaterite can be formed by tuning the cellulose concentration [17].

The results confirmed that when cellulose is in low concentrations it has a repressing impact on the transformation of vaterite to calcite, in turn promoting the formation of vaterite crystals. Vaterite immobilization onto cellulose causes an increased cytotoxic effect and a high protein adsorption capacity of cellulose.

The cytotoxicity test of the cellulose/vaterite nanocomposite was conducted using MTT assay on the cholangiocarcinoma cell line (Figure 6.1). The results indicated that the cell viabilities remained higher than 94% when the cells were cultivated with cellulose/vaterite hybrids, implying that the cellulose/vaterite nanocomposites have exceptional cytocompatibility. The hemoglobin (Hb) adsorption on the surface of cellulose/vaterite was probed at various Hb dilutions by Fourier transmission infrared (FTIR) spectroscopy as shown in Figure 6.2. The peak at 1659 cm^−1^, due to Hb fragments befallen after adsorption, indicated that the Hb has been productively adsorbed onto the vaterite/cellulose nanocomposites [17]. This implies that vaterite-laden surfaces may be coated with a variety of bioactive macromolecules (e.g. proteins, polyamino acids) for the increased specific bioactivities of such surfaces (i.e. cellular adhesion and proliferation, antimicrobial properties).

The section below describes how vaterite crystals can be grown onto a surface of microfibrillar cellulose. The initial material obtained was lignocellulose, and by acid and oxidative treatment, it was converted to microfibrillar cellulose (MFC). The crystallization of CaCO_3_ on microfibrillar cellulose surface was performed by treatment with CaCl_2_ and Na_2_CO_3_. As vaterite is a substance important in (bio)material science due to its applications in drug delivery, it is typically crystallized in bulk solution, but herein the physical immobilization approach was used to synthesize vaterite on cellulose to produce an additive-free functional vaterite coating on an organic matrix in order to increase the loading and unloading of bioagents for drug delivery.

**Figure 7:**
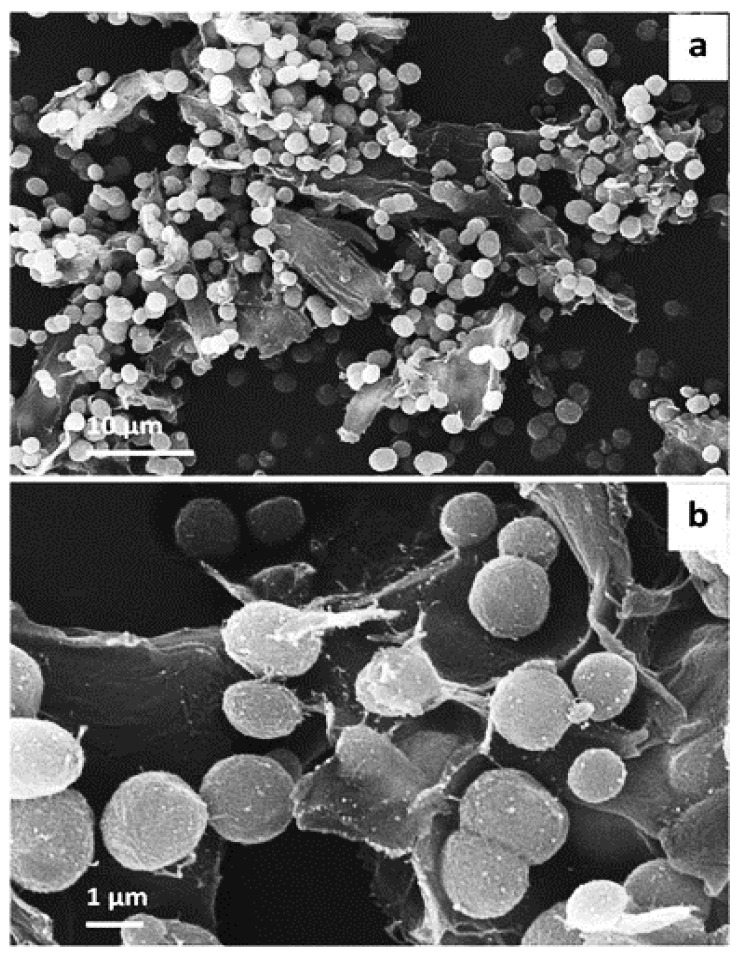
Representative SEM images showing (**a**) the overview of MFC coated with CaCO_3_ crystals in water and (**b**) enlarged surface of MFC-CaCO_3_. The Figure was adapted with permission from [32].

SEM images were obtained for CaCO_3_ crystals grown upon the surface of microfibrillar cellulose. The images demonstrate the formation of 1-2 micron-sized vaterite crystals. It is generally accepted that shape of calcium carbonate crystal refers to polymorph, thus, in this case, spherical is related to vaterite formation, along with typically high surface porosity. The smaller size of vaterite here is attributed to the effect of microfibrillar cellulose, which directed the crystallization of vaterite. The majority of the formed crystals were present on the surface of the substrate, although few of them were in bulk solution also—this may be regulated by the microfibrillar and CaCl_2_ interaction. Critically, the majority of the crystals remained adsorbed to the surface, even after multiple washing stages—which was an indication of successful solid-surface immobilization of vaterite, and modification of the surface of the substrate [32] (Figure 7).

**Figure 8:**
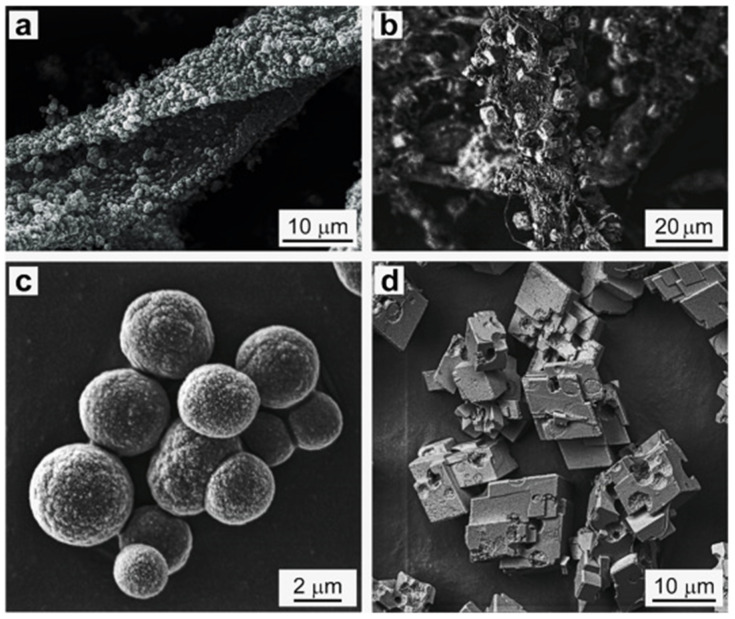
SEM images of CaCO_3_ crystals formed on the MFC surface (**a**,**b**) and CaCO_3_ crystals formed in the bulk solution (**c**,**d**). (**a**,**c**) MFC-CaCO_3_ hybrids and CaCO_3_ crystals were stored dry in the air for one week; (**b**,**d**) MFC-CaCO_3_ hybrids and CaCO_3_ crystals were incubated in water for one week. In all samples, CaCO_3_ crystals were loaded with BSA^TRITC^ via co-synthesis (C_0_ is 0.33 mg mL^−1^). This Figure was adapted with permission from [32].

The loading capacity and morphology of CaCO_3_ formed on the cellulose substrate and in bulk was compared. Vaterite-MFC hybrids were loaded with protein bovine serum albumin labelled by tetramethylrhodamine (BSA^TRITC^). The loading proficiency of BSA^TRITC^ was 70 wt.% for CaCO_3_ crystals formed in bulk, and 60 wt.% for MFC-CaCO_3,_ respectively. The encapsulation values confirm the formation of vaterite on the MFC substrate, in turn confirms the modification of MFC by vaterite growth. Along with the encapsulation efficiency, the release of BSA for CaCO_3_ and MFC-CaCO_3_ was determined, i.e., release of 60 and 55 wt.% respectively. The SEM images of BSA^TRITC^ loaded CaCO_3_ prepared in bulk and on the surface, after one week of incubation in water (Figure 8a,b) and in the dry state (Figure 8c,d) are shown. The wet samples recrystallized to calcite, with a rhombohedral structure after one week of incubation in water, as is typical for vaterite stored in aqueous solutions [33]. The CaCO_3_ and MFC-CaCO_3_ dried and kept in air remained as vaterite, even after one week. The results confirmed that microfibrillar cellulose containing vaterite CaCO_3_ crystals upon its surface is a promising template to store and release bioactive agents for drug delivery. The size and morphology of particles can be controlled by varying the MFC concentration. Thus, the modification of microfibrillar cellulose via growth of vaterite provides insight into enhanced properties of such surfaces, with the ability to actively transport bioactive substances and to form scaffolds with improved morphology. The anticipated method for the assembly of vaterite crystals upon a solid substrate (such as, for example, MFC) enables novel applications in biomedical science and opens new ways for the simple, reproducible, and up-scalable production of vaterite on surfaces. As for the future, there is a frantic need for vaterite commercialization within the biomedical and material science sector. The vaterite polymorph is a promising carrier for delivering drugs and bioactive agents for clinical applications [32]. It is of note that vaterite has successfully been utilized to assemble polymer-based structures, such as the layer-by-layer assembled capsules [34] or polymer beads (i.e., pure protein particles [35,36]). This approach is based on sequential deposition of polymers upon the surface of vaterite crystals, followed by decomposition of the vaterite matrix, and finally, the formulation of a polymer-based network (so-called capsules or particles). If pre-loaded with protein, followed by the cross-linking of such proteins; when vaterite undergoes dissolution, a pure protein particle is formed—of which replicates the inverse structure of the vaterite crystal [37,38,39,40]. Such polymer particles can be very stable and easily tuned if no significant ionic stresses take place during the dissolution of vaterite [41]. This approach allows for the encapsulation of various bioactive molecules at mild conditions with a controlled loading mechanism (e.g. proteins and enzymes as well as small drugs) [15,16,42,43,44,45,46] and demonstrates simple methods of release via external stimuli such as temperature and ionic strength [47,48] variations of which are relevant to biomedical applications such as implant coatings [49,50]. It is of note that bioactive molecules can be themselves a solid matrix treated by the layer-by-layer deposition, for instance, protein aggregates [51,52]. Temperature controlled additive-free formulation of vaterite crystals with adjusted pores and dimensions from nano- to macro-level [32,53] makes the vaterite-based encapsulation a new paradigm for controlled drug delivery via free-standing drug-loaded structures. Recently, vaterite cores have been employed for the assembly of large structures, such as tailor-made polymer-based porous scaffolds [54,55]—illustrating its use as a versatile yet affordable biomaterial for future medical and clinical use.

### 2.3. Collodion Membrane

CaCO_3_ crystals produced using the carbon dioxide dispersion approach have been paired with collodion tissue to stimulate the directional growth of such crystals. The collodion membrane directed the formation of hexagonal vaterite crystals at 28 °C. The membrane bio-mimics the organisms forming CaCO_3_, favouring the biomineralization of CaCO_3_ polymorphs. The hydroxyl group present on the collodion membrane interacts with calcium ions provided by CaCl_2_ to favour vaterite synthesis but may also yield various types of crystals [56,57].

**Figure 9:**
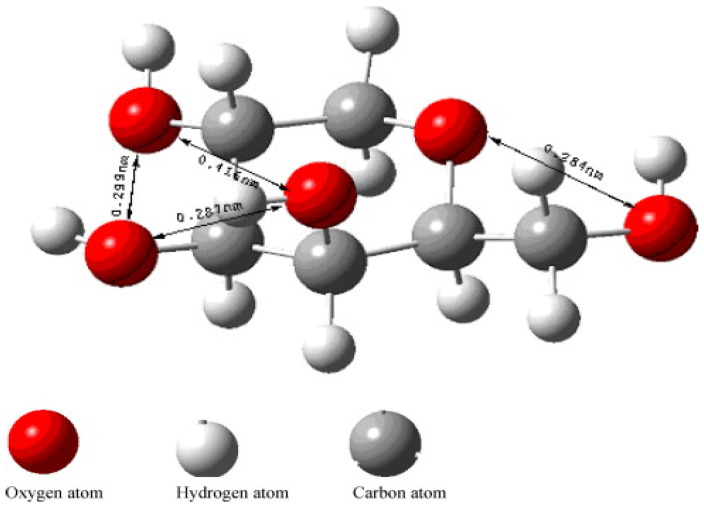
Stereo structure of a monomer produced the collodion membrane. The monomer binds to calcium ions for vaterite growth. This figure is adopted with permission from [57].

The structure of the monomer forming the collodion layer is shown in Figure 9, this monomer binds to calcium ions to guide the formation vaterite CaCO_3_. Each calcium ion has five coordination sites of which can bind to the monomer of the collodion membrane. There are five oxygen atoms providing five sites present in the monomer that can bind with these five coordination sites of calcium. The distance between one of the two oxygen atoms was determined to be 4.16 Å, and this distance matches the ab plane of vaterite which formed as result of binding of calcium on the monomer. This induces the initial formation of vaterite by the membrane as compared to other polymorphs [57].

**Figure 10:**
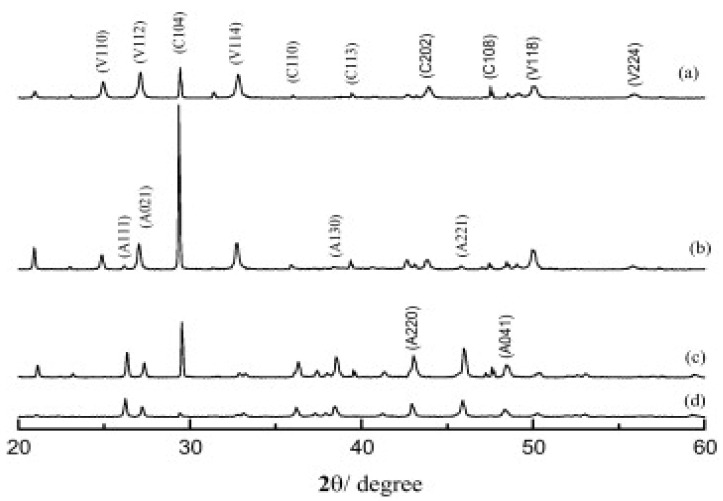
XRD patterns of the samples obtained at different temperatures: (**a**) 28 °C, (**b**) 40 °C, (**c**) 70 °C, and (**d**) 75 °C. (C: calcite; V: vaterite; A: aragonite). This Figure is adopted with permission from [57].

Different temperatures used in the growth process of CaCO_3_ affect the resultant polymorph formed, as temperature is of the many factors in the synthesis process. The XRD pattern of CaCO_3_ polymorphs obtained at different temperature are shown in Figure 10. The obtained Bragg peaks are marked with symbols A, C and V, which are related to aragonite, calcite and vaterite, respectively. At 28 °C, a mixture of calcite and vaterite formed (Figure 10a), but when temperature was increased to 40 °C, only aragonite was present (Figure 10b). The peaks corresponding to the formation of vaterite vanished when the temperature was increased to 70 °C. The further increase in temperature from 70–75 °C confirmed the co-existence of aragonite and calcite [57] (Figure 10c,d).

### 2.4. Protein Surfaces

In nature, living organisms have the ability to produce minerals with unique functionalities via biological processes. Researchers have been inspired by the structure and biomineralization activity of mollusks shells, which has led to the formation of CaCO_3_ minerals. Biomineralization includes the effect of various biomolecules, including proteins, in the growth of minerals. The proteins act as templates for the growth and nucleation of CaCO_3_ polymorphs with different morphologies. Former studies confirmed the use of single proteins for biomineralization, but the single protein does not have sufficient versatility assuring the formation of minerals. Genetic studies have confirmed that several proteins are needed to achieve the biomineralization process that occurs in natural organisms, such as mollusks’ shells. Herein, biomineralization is achieved using different proteins with different functionalities, and in turn leading to the formation of novel materials [58,59,60,61].

**Figure 11:**
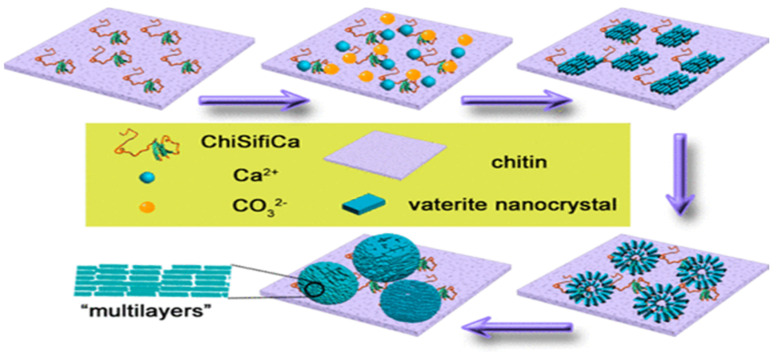
Schematic of CaCO_3_ mineralization directed by ChiSifiCa on the surface of chitin. This Figure is adapted with permission from [62].

The multifunctional protein coined ChiCaSifi was designed to induce the crystallization of CaCO_3_ polymorphs, including vaterite, on its surface. The protein ChiCaSifi contains the chitin protein (Chi) obtained from the plasmid pTWIN1, the “calcium attaching” protein is obtained from oyster—(Ca), and (Sifi) as a silk fibroin protein. Thus, it has multiple approaches for modulating the direction of CaCO_3_ formation. The chitin segment of the multicomponent protein improves the interaction between the protein and substrate. Both the chitin matrix and silk fibronin drives the crystallization of CaCO_3_ of varying morphologies. Hence, all the proteins involved have different functions and serve as a mediator for CaCO_3_ growth like that occurring naturally in mollusks. Impacts of ChiCaSifi in controlling the formation of specific CaCO_3_ polymorphs on the chitin surface were proved. Structural changes of ChiCaSifi were evidenced and related to its functions on mineralization; these observations indicate that rationally designed proteins with functional domains of mineralization proteins can be effective tools in materials synthesis. This may not only provide an insight into the formation of natural biomaterials, but also open a new avenue in the design and synthesis of novel organic–inorganic composite materials. In short, this multicomponent protein was produced using *E. coli* and was bound to the chitin matrix via a chitin binding protein in its structure. The chitin matrix absorbed the calcium ions present with the aid of the calcium binding protein in ChiCaSifi, and the mineralization begins. The alpha helix of silk fibroin protein stabilizes the as prepared CaCO_3_. Herein, when the binding interaction between ChiCaSifi and chitin was increased, the formation of vaterite was observed on the chitin surface. The biomineralization process of CaCO_3_ intervened by ChiCaSifi on the chitin surface [62] is illustrated in Figure 11.

**Figure 12:**
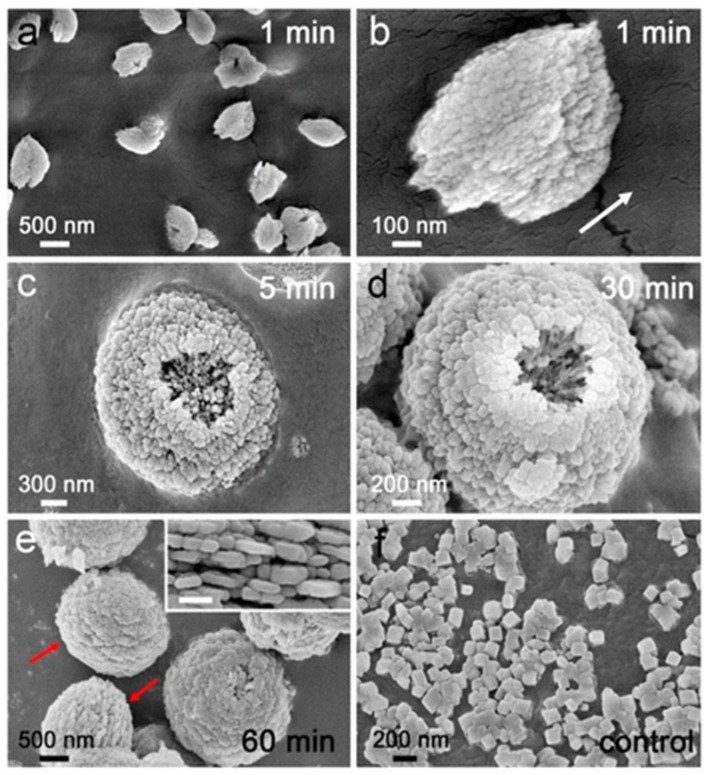
SEM images of CaCO_3_ minerals on the surface of chitin. (**a**) Low and (**b**) high magnification of minerals obtained in 1 min of mineralization, (**c**) 5 min, (**d**) 30 min, (**e**) 60 min. (**e**, inset) The equator of mineral. Scale bar in the inset is 200 nm. The equator of the minerals was pointed by red arrows. (**f**) Minerals obtained in the absence of protein. The figure is adapted with permission from [62].

The attachment of ChiSifiCa on the surface of chitin provides a microenvironment to induce the nucleation and growth of CaCO_3_ and shuttle-like CaCO_3_ minerals observed (Figure 12a). The shuttle-like minerals were assembled by nanoparticles (Figure 12b), as is typical for vaterite. The interior alignment of nanoparticles was well oriented as illustrated by the direction of arrow, but some holes has been shown indicating that internal nanoparticles remained liquefied. The size of CaCO_3_ minerals and holes increased to micrometer with semi-sphere morphology of particles within 5 min of reaction (Figure 12c). Within 30 min of mineralization reaction, the holes were enclosed by assembly of nanoparticles on top of the semi-spherical minerals - leading to the formation of spherical shaped vaterite particles (Figure 12d). Within 60 min of reaction time, the intact spherical vaterite minerals were formed (Figure 12e). Therefore, this study demonstrated that the protein ChiCaSifi plays an important role in the formation of vaterite, as it was the preferred polymorph in this process. Hence, multicomponent proteins can be designed according to their specific functions for use as biomineralization tools [62].

Fibrin is the protein by-product of blood coalescence, and it was found to be a solid substrate favoring the immobilization of vaterite on its surface. The vaterite crystals were grown on the surface of fibrin by the constant composition technique; the supersaturated CaCO_3_ solutions were used. Fibrin stabilizes the CaCO_3_ polymorph and prevents the transformation of vaterite to calcite. High supersaturation along with experimental conditions of a temperature of 25 °C and a constant pH of 8.5, increased the stability of CaCO_3_ and thus prevents the phase transformation from vaterite to calcite [63].

The crystallization of vaterite on fibrin was confirmed by SEM, FTIR spectroscopy analysis and XRD patterns. SEM was used to study the morphology of immobilized vaterite crystals onto fibrin. The SEM image of the fibrin substrate and spherical vaterite crystals is shown on fibrin are presented in Figure 13.1,2. The FTIR spectrum exhibited the characteristic absorption peaks for vaterite formation at 1480, 1070, 873, 848 and 795 cm^−1^ respectively (Figure 13.2). The XRD spectrum exhibited characteristic hkl values, confirming the formation of vaterite on the fibrin surface (Figure 13.3). The results therefore confirmed the immobilization of vaterite on fibrin [63].

### 2.5. Polymer Grafted Surfaces

**Figure 14:**
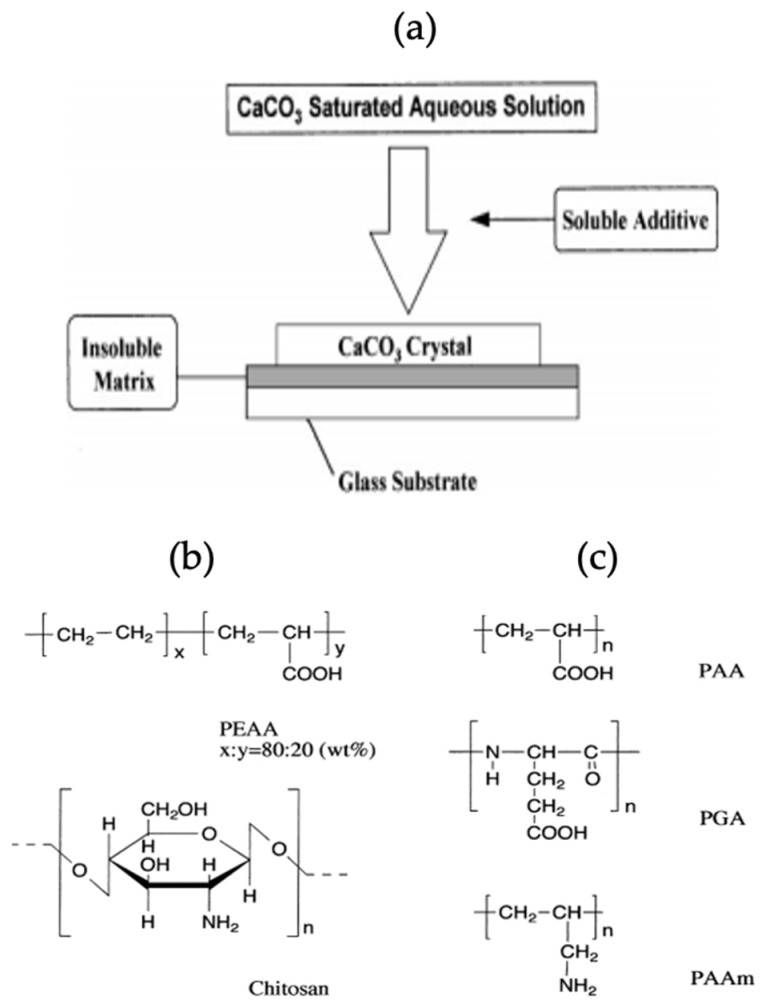
(**a**) Scheme for the crystallization process of CaCO_3_ in the presence of soluble additives and insoluble matrices, (**b**) Structures of insoluble matrices and (**c**) Structures of soluble additives. This Figure is adapted with permission from [64].

Biominerals show association with biomolecules due to the presence of specific functional groups leading to the synthesis of mineral polymorphs. The biomolecules such as proteins and polypeptides control the size of particles formed, as well as the morphology of particles immobilized on their surface. One study shows the effect of an insoluble matrix and soluble organic additives on the crystallization of CaCO_3_. The proper use of polymer items for the handling of inorganic substances results in the formation of a modern class of organic/inorganic materials which have distinctive properties. The effect of water-soluble macromolecules as soluble additives on the crystallization of CaCO_3_ and the collaborative impact of soluble additives and insoluble matrices on the formation of CaCO_3_ crystals is pronounced. The crystallization scheme of CaCO_3_, structure of soluble additives and insoluble matrix used in this study have been shown schematically in Figure 14 [59].

**Figure 15:**
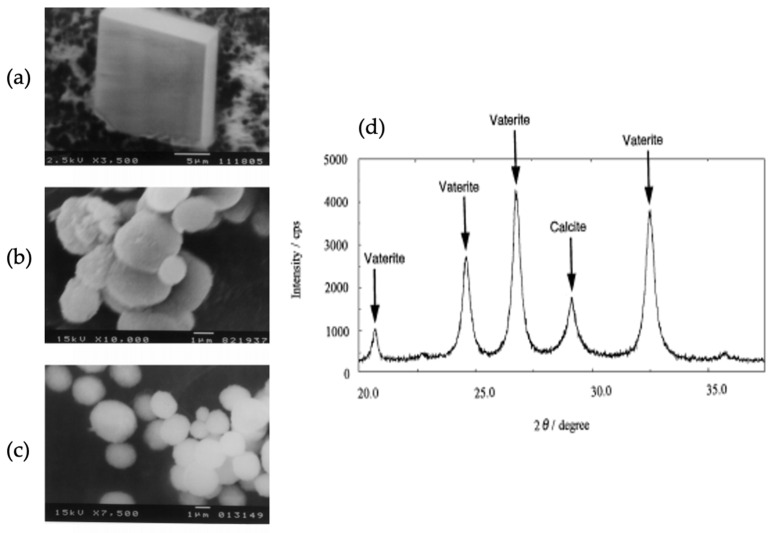
SEM micrographs of CaCO_3_ crystals grown on a glass substrate; (**a**) in the absence of macromolecules; (**b**) in the presence of PGA; (**c**) in the presence of DNA. (**d**) X-ray diffraction pattern of CaCO_3_ crystals. Soluble additive: PGA, 5.0 × 10^−3^ wt.%. Insoluble matrix: none. This Figure is adapted with permission from [64].

CaCO_3_ grown on a glass substrate, coated with insoluble matrix resulted in the formation of three polymorphs of CaCO_3_, and the morphology of the prepared crystals has been observed using SEM. The SEM images confirmed that in the absence of a soluble additive and an insoluble matrix, the rhombohedral calcite crystals are formed on a glass substrate (Figure 15a). The use of polyacrylic acid (PAA) or polyacrylic amide (PAAm) as soluble additives leads to no crystal growth, as PAA/PAAm inhibit the immobilization of CaCO_3_ on the glass substrate. When the glass substrate had adsorbed (polyglutamic acid) PGA/DNA (deoxyribonucleic acid), spherical vaterite crystals were grown [64] (Figure 15b,c). The XRD diffractogram of the CaCO_3_ polymorph obtained on glass adsorbed with PGA confirmed the co-existence of calcite and vaterite (Figure 15d) and no crystallization was observed in the presence of PAA. The PAA has the same carboxylic acid fraction as PGA but gave different results. Thus, it has been confirmed that the configuration of the polymer has an influence on the crystallization process [64].

The cooperative effect of soluble additives and insoluble matrices was studied by choosing poly (2-ethylacrylic acid) and chitosan as an insoluble matrix, coated on a glass substrate. In the presence of insoluble matrices without additives, the rhombohedral calcite polymorph was grown. CaCO_3_ was then prepared in the presence of soluble additives containing functional groups; when PAA/PAAm were used as additives there was no crystallization of CaCO_3_, whereas, in the presence of DNA as a soluble additive, spherical vaterite was formed on the substrate. Remarkably, CaCO_3_ crystals grew on the chitosan matrices when polyacrylic acid/polyglutamic acid (PAA/PGA) were used. The effect of PAA and PGA as soluble additives on immobilization of crystals is different when the CaCO_3_ crystallization is performed on the glass substrate coated with poly (2-ethylacrylic acid) or chitosan. Though, on the chitosan matrices, both polymers PAA/PGA led to the formation of a thin film of CaCO_3_, due to the interaction of the carboxylic group of the polymers and the amino group of chitosan. The inorganic/organic composites, which can be referred to as hybrid structures [65], stated here may provide new biomimetic tools with specialized mechanical and biological properties [64].

**Figure 16:**
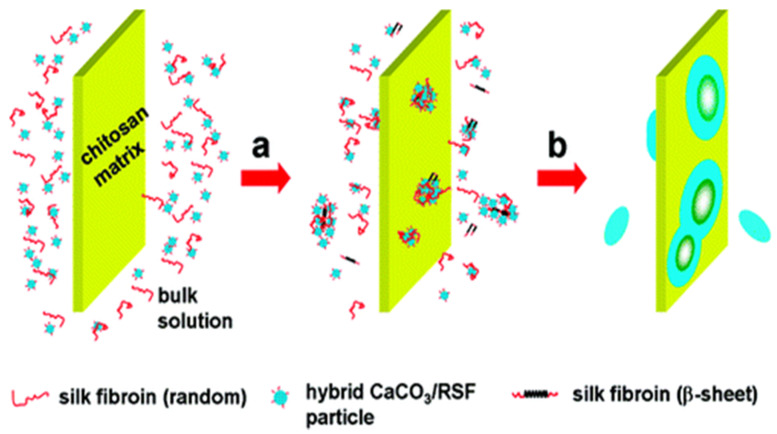
Mechanism of the formation of disk-like CaCO_3_: (**a**) nucleation of CaCO_3_ due to the adsorption of RSF and CaCO_3_/RSF hybrid nanoparticles; (**b**) accumulation of CaCO_3_/RSF hybrid nanoparticles inducing growth of the disk-like crystals. The figure is adapted with permission from [68].

The synthesis of vaterite was studied using the biomineralization approach and the with a chitosan substrate containing regenerated silk fibroin (RSF). This biomineralization system resembles the natural system of molluscs, wherein chitosan, the alternative of chitin, and regenerated silk fibroin resemble the silk protein present in pearl shells. Indeed, RSF controlled the CaCO_3_ crystallization on the chitosan surface (substrate). By controlling the pH and temperature, different CaCO_3_ polymorphs can be formed on the surface of chitosan; this is typical for the controlled precipitation of various CaCO_3_ polymorphs [66,67]. The process of nucleation starts with the hydrophobic aggregation of RSF; CaCO_3_ is attracted to the RSF via electrostatic forces. The RSF/CaCO_3_ hybrid formed initially was adsorbed onto the chitosan substrate by hydrogen bonding between -CONH and -NH_2_ groups, and this leads to vaterite growth. The continuous nucleation leads to the formation of disc like vaterite crystals. The possible mechanism has been shown (Figure 16) [68].

**Figure 17:**
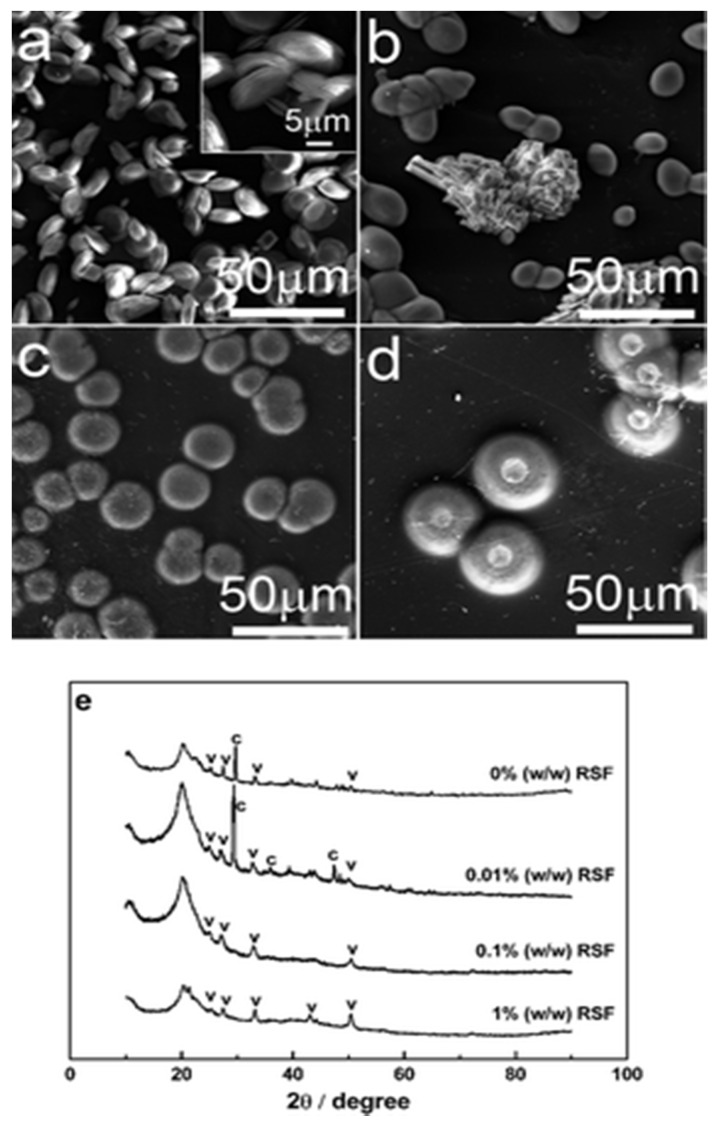
CaCO_3_ products grown on chitosan membranes with a range of concentrations of RSF at initial pH 7.9 for 1 day. (**a**) 0% (*w*/*w*), (**b**) 0.01% (*w*/*w*), (**c**) 0.1% (*w*/*w*), (**d**) 1% (*w*/*w*), and (**e**) XRD patterns of CaCO_3_ products above. “c” = calcite; “v” = vaterite. This figure was adapted with permission from [68].

The silk fibroin influences crystal growth; SEM and XRD studies showed that RSF influenced the morphology and polymorphism of CaCO_3_ particles. At low pH calcite was formed, while spherical vaterite was formed at elevated pH. Results demonstrated that without silk fibroin in the solution there were more vaterite crystals, and they transformed into calcite within a week (Figure 17a,e). However, at 0.01% RFS, vaterite of ellipsoidal shape and rhombic calcite were grown on the chitosan substrate (Figure 17b,e), and when the concentration was increased to 0.1% and 1% *w*/*w*, only the vaterite form was present (Figure 17c,e) at a pH value of 7.9 [68], as supported by XRD. Such results confirmed the formation of vaterite crystals on the surface of chitosan membrane assisted by regenerated silk fibroin protein [68].

### 2.6. Fibres

Previous studies confirmed that organic matrices consist of a soluble and insoluble matrix; the soluble part is provided by anionic biomolecules such as peptides and proteins. The insoluble matrix is formed by hydrophobic scaffolds like collagen and chitin,and leads to the nucleation of CaCO_3_ polymorphs. The soluble biomolecules adsorbed onto the insoluble matrix surface can act as a scaffold for the immobilization of CaCO_3_ on the surface of the biological substrate. Herein, this study demonstrated the participation of the functional groups of polyamide fibres present on the insoluble matrix surface for growth of CaCO_3_. The insoluble polyamide matrix contained polyalanine and polyglycine in their structure for initiating the growth of vaterite. This matrix also contains domains of Nylon 66 and Kevlar 29 in their lattice structure. The carboxylic groups and amine groups present favored the growth of vaterite particles in the presence of the water soluble additive polyvinyl alcohol. The functional groups of polyamide promote the nucleation of calcite, but here, with the addition of poly(vinyl alcohol), the selectivity was enhanced. The unmodified (without acid/base treatment) polyamide with PVA favoured vaterite nucleation and the chemically modified form of polyamide with PVA favoured aragonite nucleation. The modified polyamide (with acid/base treatment) favoured the formation of aragonite. Untreated polyamide adsorbed with PVA gave 81% vaterite, along with calcite on the surface of Nylon 66 and Kevlar 29 (polyamide fibres). Morphological characterization of the polymorphs was done using SEM, and the nature of the crystal lattice was identified using XRD. The XRD pattern and SEM micrographs confirmed that a higher concentration of PVA gave only the vaterite polymorph (Figure 18.1) with a minor amount of calcite [69] (Figure 18.2).

### 2.7. Extracellular Matrix Substances

**Figure 19:**
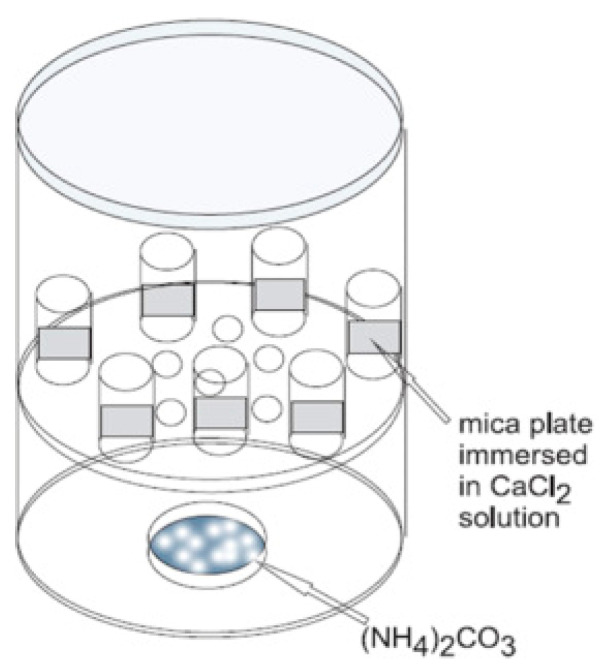
Schematic representation of experimental system for calcium carbonate mineralization. The figure is adapted with permission from [29].

**Figure 20:**
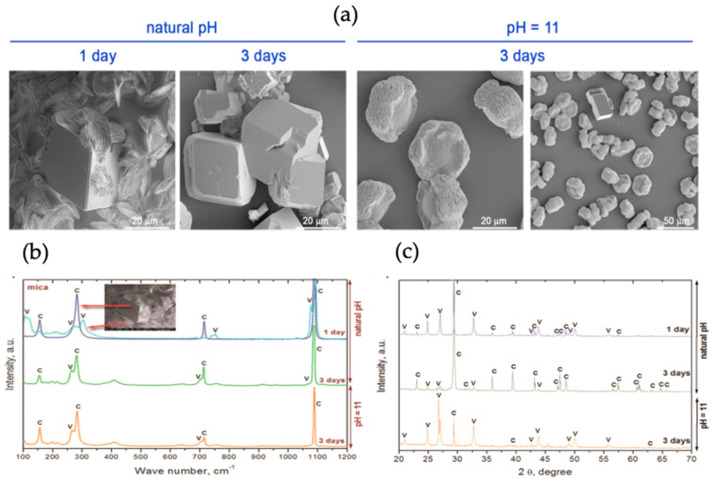
(**a**) Representative SEM images of calcium carbonate microcrystals deposited on the mica surface at natural and basic initial pH, (**b**,**c**) Raman spectra and XRD spectra of calcium carbonate deposited on bare mica (C-calcite, V-vaterite). The figure is adapted with permission from Reference [29].

The effect of the extracellular polymeric substance (EPS) layer obtained from *Rhodococcus opacus* bacterial strain was studied on the morphology and growth of CaCO_3_ on the mica substrate. Different fractions of EPS were spread on the mica surface via spin coating to observe the influence on CaCO_3_ synthesis. Vaterite (as well as silica) micro- and nanoparticles have been used to functionalize the substrates using spin-coating [70]. H. Riegler has developed a theory of how particles are adsorbed upon spin-coating. The vapour diffusion method was adopted for the nucleation of CaCO_3_ on mica assisted by EPS. The schematic representation of CaCO_3_ mineralization is shown in Figure 19. The mica slides were immersed in plastic boxes containing CaCl_2_ solution and, NaOH was used to stabilize pH. The plastic boxes were kept in a desiccator containing NH_4_CO_3_ to complete the synthesis of CaCO_3_. SEM was used to study the morphology of formed crystals, Raman spectroscopy and XRD patterns were taken to determine the polymorphs produced [29], as shown in Figure 20.

The results were obtained after 1 and 3 days of synthesis at natural initial pH and at pH 11, respectively (Figure 20a). At the initial pH, the rhombohedral (calcite) and lens shaped vaterite crystals were obtained; with a longer period of crystallization, rhombohedral crystals with altered structure were formed. This was an indication of the phase transfer from more soluble polymorphs to less soluble forms, i.e., calcite. The crystals obtained at pH 11 were rosetta shaped, indicating the synthesis of vaterite [29], a similar structure was observed in previous studies [71,72]. The results obtained from XRD and Raman spectroscopy confirmed the presence of a mixture of calcite and vaterite on the mica substrate (Figure 20b). The results demonstrated that after one day at natural pH, 83% vaterite was present and, after three days, 18% remained, but the three-day growth of vaterite at pH 11 gave 93% of vaterite polymorph. These results indicate that the water-soluble fraction of EPS fluid favoured vaterite dissolution and calcite nucleation, and the total EPS stabilizes vaterite at basic pH. Hence, the crystal size and polymorphic form CaCO_3_ can be controlled by the EPS obtained from *Rhodococcus opacus,* and by of the functional groups present in EPS fractions [29].

Layer-by-Layer (LbL) assembled polymer networks (so-called multilayers) can serve as an artificial extracellular matrix being deposited at solid surfaces; this allows for the inclusion of multiple components to achieve multifunctional properties that are indispensable for mimetics/regulation of dynamic and complex biological structures [73,74,75,76,77]. Such multilayers are complex structures with their internal structures and formation mechanisms still under investigation. Understanding the activity of biomacromolecules as their components is essential [78,79] in the LbL films, which have been employed as the main component of multilayer capsules that ensures capsule stability, biocompatibility, and a broader application range [80]. They can be used in future for the controlled assembly of vaterite crystals at solid surfaces, forging a new direction in this research field.

### 2.8. Microbiologically Induced Vaterite Formation

Several microorganisms are involved in the mineralization of CaCO_3_. The process of bacteria induced CaCO_3_ precipitation has received a lot of consideration from researchers working in various disciplines. Consequently, one of the most studied subjects is the advancement in technologies the use microorganisms to induce the formation of CaCO_3_ polymorphs to restore concrete structures [81]. Findings on the stability and formation of bacterially induced CaCO_3_ has gained importance for the understanding of the mechanism of CaCO_3_ mineralization.

Biogenic vaterite has greater stability than chemically synthesized vaterite due to the organic material produced by bacteria which enwrapped vaterite and prevents its transfer to more stable polymorphs [82]. This is also observed in synthetic vaterite synthesis with the addition of LbL coatings and polymer matrices [46,83,84]. Recent findings suggest that bacteria might induce the formation of polymorphs of CaCO_3_ serving as templates, and that the carbonic anhydrase, also produced by themselves, plays an essential role in the mineralization of vaterite CaCO_3_ [85]. Bacterially induced CaCO_3_ mineralization is thought to be important in a range of processes and the study [85] indicates that *Myxococcus xanthus*, a soil bacterium, facilitates the formation of spherical vaterite CaCO_3_. The production of CO_2_ and NH_3_ by bacteria, the transformation of NH_3_ to NH_4_^+^, and the presence of OH^−^, leads to increased basicity, leading to the physiochemical conditions favourable for vaterite precipitation onto the surface of bacteria [86]. Past findings also indicate that bacterial species like Actinomycetes play an important role in the formation of mineral deposits in caves [87].

*Lysinibacillus sphaericus, Raoultella planticola*, and *Streptomyces pluricolorescens* are proficient in inducing CaCO_3_ polymorphs through a nitrate reduction, both anaerobically and aerobically. The produced CaCO_3_ crystals were analysed using SEM, EDX and XRD and the results confirmed that the biogenic bacteria serve as nucleation sites for the precipitation of CaCO_3_, having distinctive shape and morphology. Hence, the applications of these bacterial strains that have been used in CaCO_3_ mineralization bode well in the biomedical and environmental fields [88]. Intriguingly, the scientific relevant literature has reported aragonite and calcite as the most common bacterial carbonates, while this analysis observed only calcite and vaterite in all tested samples; this can suggest that precipitation of vaterite might be more common than expected by rather quick recrystallisation of vaterite to calcite in aqueous media [89].

Some studies have indicated that microbiologically induced CaCO_3_ precipitation, with some changes in surface characteristics and pH, can lead to the formation of biofilms [90]. Researchers have found that nanomechanical and morphological properties of CaCO_3_ can be tailored if they are precipitated via bacteria. Due to these properties, bacteria induced CaCO_3_ polymorphs are being employed for engineering, biomedical and geological applications [91,92].

## 3. Conclusions

This review focuses on an overview of methods used to synthesize vaterite crystal on solid/organic surfaces. CaCO_3_ typically exists as three anhydrous polymorphic forms, but here, vaterite is the material of choice due to the numerous and increasing applications of this carbonate in material science. Biomolecules can assist in the biomineralization of CaCO_3_ to result in the fabrication of bioinspired materials which mimic the natural phenomenon of biomineralization. The methods demonstrated explain the immobilization of vaterite by both physical and chemical approaches. Physical immobilization approaches have been widely studied, as this process is facile to achieve. Whereas chemical immobilization involves strong bonding between biomolecules and the vaterite crystals, which in turn leads to irreversible changes of the surface/molecule. The physical immobilization approach is used more commonly as this does not require additional modification of biomolecules and coupling reagents as with chemical immobilization. This makes the physical approach to be an economically preferential process as compared to the chemical approach. Multiple studies reported the synthesis of vaterite on biomaterials modified with peptides, proteins, polysaccharides, and biopolymers. The growth of small nanometer-sized vaterite crystals leads to modification of biomolecular surfaces, as the smaller sized particles have greater surface area, and their properties and activities may be dramatically enhanced. The biomolecules containing vaterite on their surface can act as a carrier of bioagents as, for instance, the microfibrillar cellulose after the growth of vaterite on its surface may act as a protein delivery vehicle. The loading and unloading of bioagents on modified biomolecules can be done to achieve better scaffolds for controlled and programmed drug and protein delivery. Vaterite offers enormous possibilities for the encapsulation and release of bioactives of different nature that may include not only large biomacromolecules but even small anticancer drugs, lipids, antimicrobials and other relevant molecules [93,94,95,96,97,98,99]. There is limited study on this aspect where vaterite is formed on the solid surface of biomaterials, and we believe this relatively unexplored field will pave-way for the future fabrication of efficient biomaterials with tailor-made properties and excellent performance for various bio-applications.

## Figures and Tables

**Figure 6:**
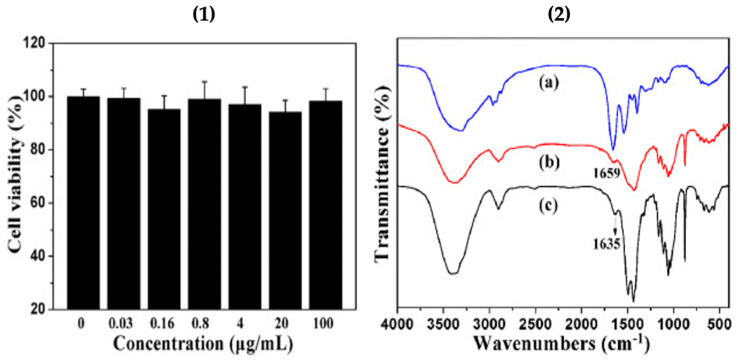
(**1**) Cytotoxicity tests of the cellulose/vaterite nanocomposites, and (**2**) FTIR spectra of pure Hb (**a**) and the cellulose/vaterite nanocomposites after (**b**) and before (**c**) Hb adsorption. This Figure is adapted with permission from [17].

**Figure 13:**
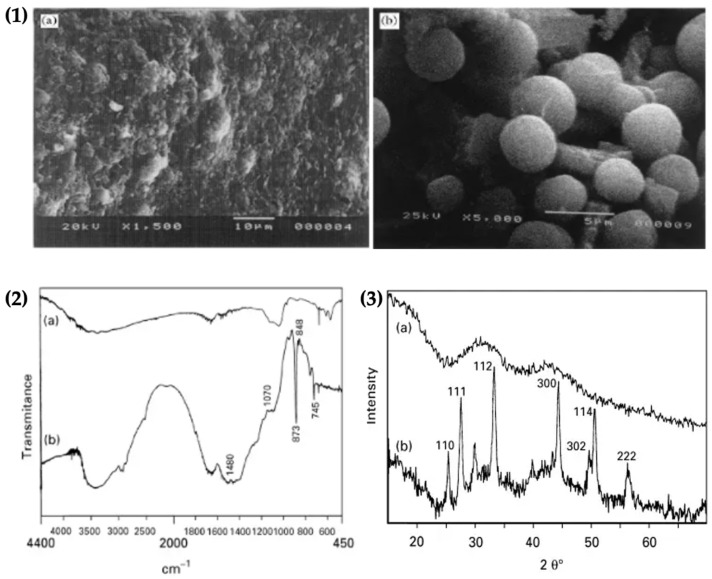
(**1**) Scanning electron micrographs: (**a**) fibrin substrate; (**b**) vaterite crystals on fibrin, Ca5 "3]10~3M, pH"8.5, 253C; (**2**) FTIR spectra of: (**a**) fibrin; (**b**) vaterite grown on fibrin, and (**3**) Powder X-ray diffraction spectra of: (**a**) fibrin; (**b**) vaterite grown on fibrin. Adopted with permission from [63].

**Figure 18:**
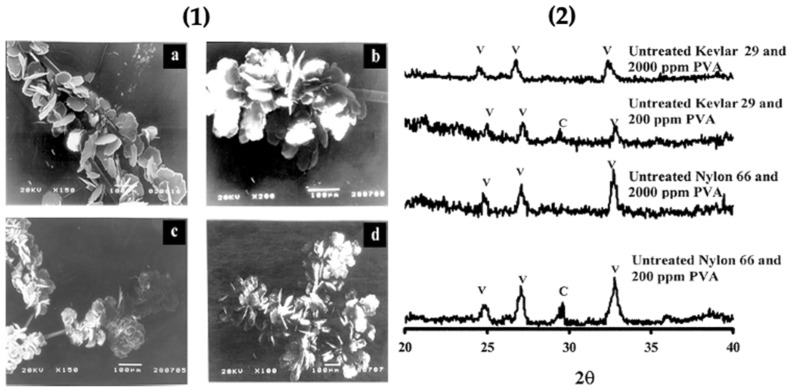
(**1**) Representative SEM images of the vaterite phase grown on untreated Nylon 66 in the presence of 200 ppm (**a**) and 2000 ppm PVA (**b**); untreated Kevlar 29 in the presence of 200 ppm (**c**) and 2000 ppm PVA (**d**); and (**2**) Representative powder XRD patterns of the calcium carbonate polymorphs formed on untreated fibres in the presence of PVA. The diffraction peaks that correspond to calcite and vaterite phases are indicated by C and V, respectively. Taken with permission from reference [69].
